# 1078. Vaccine-induced antibody level predicts the clinical course of breakthrough infection of COVID-19 caused by delta and omicron variants: a prospective observational cohort study

**DOI:** 10.1093/ofid/ofac492.919

**Published:** 2022-12-15

**Authors:** Yong Chan Kim, Bongyoung Kim, Nak-Hoon Son, Namwoo Heo, Yoon Soo Park, Heejung Kim

**Affiliations:** Department of Internal Medicine, Division of Infectious disease, Yongin Severance Hospital, Yonsei University College of Medicine, Yongin, Kyonggi-do, Republic of Korea; Department of Internal Medicine, Hanyang University College of Medicine, Seongdong-gu, Seoul-t'ukpyolsi, Republic of Korea; Department of Statistics, Keimyung University, Daegu, Taegu-jikhalsi, Republic of Korea; Yongin Severance Hospital, Yongin, Kyonggi-do, Republic of Korea; Department of Internal Medicine, Division of Infectious disease, Yongin Severance Hospital, Yonsei University College of Medicine, Yongin, Kyonggi-do, Republic of Korea; Department of Laboratory Medicine, Yongin Severance Hospital, Yonsei University College of Medicine, Yongin, Kyonggi-do, Republic of Korea

## Abstract

**Background:**

This study aimed to determine the effect of vaccine on clinical course of delta and omicron variant infection. Furthermore, we tried to evaluate the utility of antibody level against spike protein as a predictor of disease course of COVID-19 in vaccinated patients.

**Methods:**

Between December 11, 2021 and February 10, 2022, we performed a prospective observational cohort study in an institution of South Korea. Among adult patients admitted due to COVID-19, individuals with confirmed delta and omicron variant infection were included. Multivariable logistic regression analysis was performed to determine the association between antibody level and clinical course of breakthrough infection in vaccinated patients. The relationship between antibody level and cycle threshold (Ct) values was confirmed using a generalized linear model. We used the antibody titers collected within 7 days of symptom onset or diagnosis and the Ct values tested on days 5-7 days after initial diagnosis.

**Figure 1.**
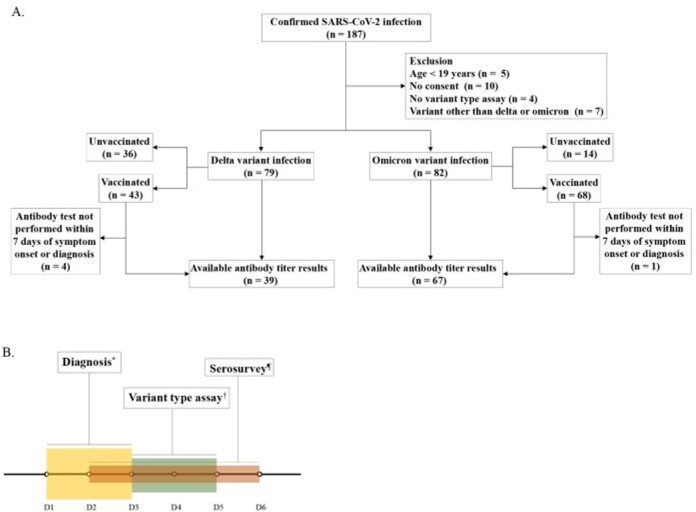
Study flow diagram. A, Study flow of enrollment. B, Severe acute respiratory syndrome coronavirus 2 diagnosis, antibody to spike protein test, variant type assay

**Results:**

Of 161 patients with delta and omicron variant infection, 106 vaccinated patients (39 delta and 67 omicron) had available serum samples. The geometric mean titers of antibodies in patients who experienced the fever (≥37.5°C), hypoxia (≤94% of SpO_2_), pneumonia, C-reactive protein (CRP) elevation ( >8 mg/L) or lymphopenia (< 1,100 cells/μL) during hospitalization were 1201.5 U/mL, 98.8 U/mL, 774.1 U/mL, 1335.1 U/mL, and 1032.2 U/mL, respectively, which were lower compared with those who did not (p< 0.05 for all). Increase in antibody level of vaccinated patients with delta and omicron infection was associated with decrease in occurrence of fever (aOR, 0.23; 95% CI, 0.12-0.51), hypoxia (aOR, 0.23; 95% CI, 0.08-0.7), CRP elevation (aOR, 0.52; 95% CI, 0.29-0.0.94), and lymphopenia (aOR, 0.57; 95% CI, 0.33-0.98) during hospitalization, regardless of virus type or booster vaccination status. Data from 33 patients who had Ct values suitable for analysis showed a positive correlation between antibody levels and Ct values (p=0.02).

**Table 1.**
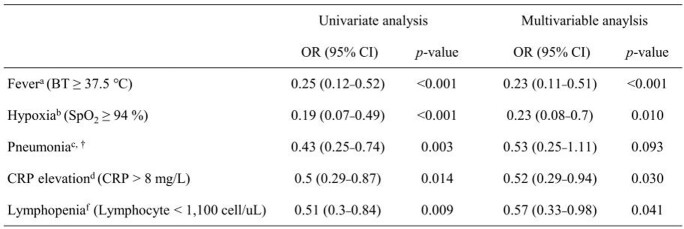
Association of antibody titres and variables with clinical courses during hospitalisation in vaccinated patients with breakthrough infections caused by delta and omicron variants

**Figure 2.**
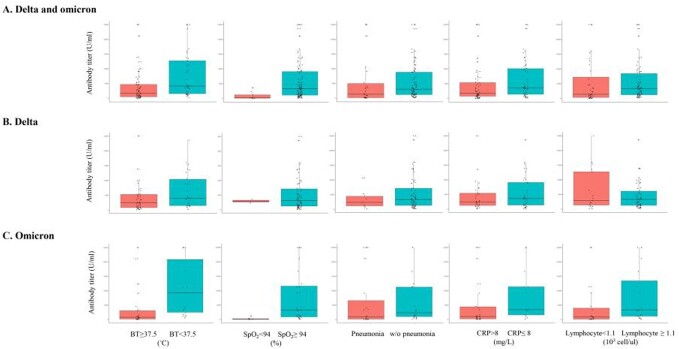
Comparison of antibody levels between vaccinated patients with or without specific signs during hospitalisation

This analysis included 106 patients with delta and omicron variant infections whose serum samples were collected within 7 days of symptom onset or diagnosis. Antibody levels are described as box plots of medians with interquartile ranges.

BT, body temperature; SpO2, percutaneous oxygen saturation; w/o, without; CRP, C-reactive protein

**Figure 3.**
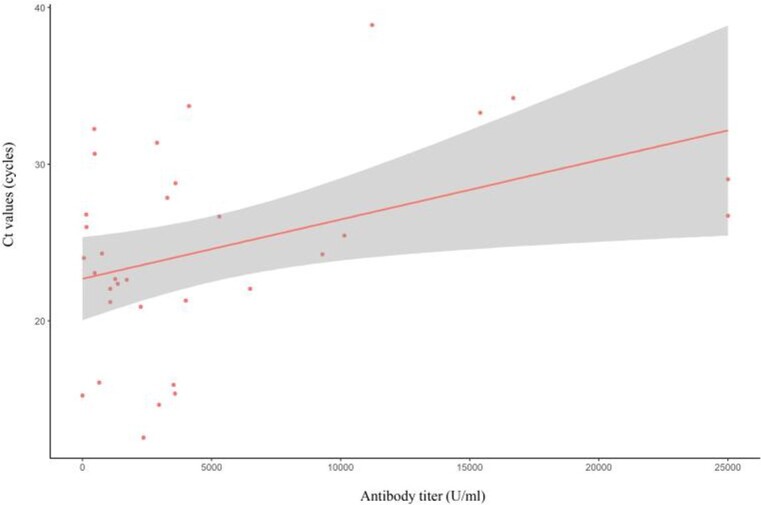
Association of antibody titres and Ct values

**Conclusion:**

Antibody levels are predictive of the clinical course of COVID-19 in vaccinated patients with delta and omicron variant infections. Our data highlight the need for concentrated efforts to monitor patients with SARS-CoV-2 infection who are at risk of low antibody levels.

**Disclosures:**

**All Authors**: No reported disclosures.

**Figure 1.** Study flow diagram. A, Study flow of enrollment. B, Severe acute respiratory syndrome coronavirus 2 diagnosis, antibody to spike protein test, variant type assay

*Patients were diagnosed at a median of 2 days (interquartile range [IQR] 1–3) after symptom onset.

¶Antibody tests were performed at a median of 4 days (IQR 2–6) after symptom onset or diagnosis, whichever was earlier.

† Variant-type assays were conducted at a median of 4 days (IQR 3–5) after diagnosis.

**Table 1.** Association of antibody titres and variables with clinical courses during hospitalisation in vaccinated patients with breakthrough infections caused by delta and omicron variants

Multivariable logistic regression analysis was performed to determine the effect of antibody levels on the clinical course of breakthrough infections. Fever, hypoxia, pneumonia, CRP elevation, or lymphopenia were selected as variables representing the clinical course. Confounding factors were included in each multivariable model, as described below. Antibody titres were log10 transformed for analyses. Abbreviations: OR, odds ratio; CI, confidence interval; BT, body temperature; SpO2, percutaneous oxygen saturation; CRP, C-reactive protein; CCI, Charlson comorbidity index.

a Multivariable analysis adjusted for age and sex.

b Multivariable analysis adjusted for age, sex, immunocompromised status, and variant type.

c Multivariable analysis adjusted for age, sex, immunocompromised status, CCI, and variant type‡

d Multivariable analysis adjusted for sex‡ and variant type.

f Multivariable analysis adjusted for sex, CCI, and variant type.

†Presence of pneumonia was determined using chest radiography.

‡ In addition to antibody titre, the omicron variant was associated with a decrease in the occurrence of pneumonia (OR, 0.150; 95% CI, 0.050-0.448); females were associated with a decrease in the occurrence of CRP elevation (OR, 0.431; 95% CI, 0.194-0.955).

**Figure 3.** Association of antibody titres and Ct values

Data from 33 patients, with Ct values measured 5–7 days after diagnosis, showed a positive correlation between antibody levels and Ct values (slope: 0.0004, p=0.022)

Abbreviations: Ct, cycle threshold; PCR, polymerase chain reaction

